# The Feasibility of an Exercise Intervention in Males at Risk of Oesophageal Adenocarcinoma: A Randomized Controlled Trial

**DOI:** 10.1371/journal.pone.0117922

**Published:** 2015-02-23

**Authors:** Brooke M. Winzer, Jennifer D. Paratz, Jonathan P. Whitehead, David C. Whiteman, Marina M. Reeves

**Affiliations:** 1 Burns, Trauma and Critical Care Research Centre, School of Medicine, The University of Queensland, Brisbane, Australia; 2 Metabolic Medicine Group, Mater Research Institute University of Queensland, Brisbane, Australia; 3 Cancer Control Group, QIMR Berghofer Medical Research Institute, Brisbane, Australia; 4 Cancer Prevention Research Centre, School of Public Health, The University of Queensland, Brisbane, Australia; University Hospital Llandough, UNITED KINGDOM

## Abstract

**Objective:**

To investigate the feasibility and safety of a 24-week exercise intervention, compared to control, in males with Barrett’s oesophagus, and to estimate the effect of the intervention, compared to control, on risk factors associated with oesophageal adenocarcinoma development.

**Methods:**

A randomized controlled trial of an exercise intervention (60 minutes moderate-intensity aerobic and resistance exercise five days/week over 24 weeks; one supervised and four unsupervised sessions) versus attention control (45 minutes stretching five days/week over 24 weeks; one supervised and four unsupervised sessions) in inactive, overweight/obese (25.0–34.9 kg/m^2^) males with Barrett’s oesophagus, aged 18–70 years. Primary outcomes were obesity-associated hormones relevant to oesophageal adenocarcinoma risk (circulating concentrations of leptin, adiponectin, interleukin-6, tumour necrosis factor-alpha, C-reactive protein, and insulin resistance [HOMA]). Secondary outcomes included waist circumference, body composition, fitness, strength and gastro-oesophageal reflux symptoms. Outcomes were measured at baseline and 24-weeks. Intervention effects were analysed using generalised linear models, adjusting for baseline value.

**Results:**

Recruitment was difficult in this population with a total of 33 participants recruited (target sample size: n = 80); 97% retention at 24-weeks. Adherence to the exercise protocol was moderate. No serious adverse events were reported. A statistically significant intervention effect (exercise minus control) was observed for waist circumference (-4.5 [95% CI -7.5, -1.4] cm; p < 0.01). Effects on primary outcomes were not statistically significant.

**Conclusion:**

This small, exploratory trial provides important information to inform future trial development including recruitment rates and estimates of effect sizes on outcomes related to oesophageal adenocarcinoma risk. Future trials should investigate a combined dietary and exercise intervention to achieve greater weight loss in this population and relax inclusion criteria to maximize recruitment.

**Trial Registration:**

Australian New Zealand Clinical Trials Registry (ANZCTR) ACTRN12609000401257

## Introduction

During the past three decades, the incidence of oesophageal adenocarcinoma has risen by over 300% in females and 500% in males in Western countries [1–5]. The incidence of the precursor lesion, Barrett’s oesophagus is also rising rapidly [6,7]. People diagnosed with Barrett’s oesophagus have an oesophageal adenocarcinoma risk of approximately 30–40 times higher than that of a healthy population [8,9]. Males are particularly at risk of oesophageal adenocarcinoma, with a male to female ratio of at least 5:1 [1]. If Barrett’s oesophagus does progress to adenocarcinoma, prognosis is poor with a 5-year survival rate of 10–15%, despite surgery and chemotherapy [10,11]. At present there is limited evidence regarding lifestyle interventions aimed at reducing cancer risk in males with Barrett’s oesophagus [12].

Lifestyle based interventions such as physical activity have the potential to reduce the risk of oesophageal adenocarcinoma given that risk factors include overall adiposity [13–17], and in particular visceral obesity [18–21]. To date, studies on the association between physical activity and oesophageal adenocarcinoma incidence have been limited, with two prospective cohort studies showing that increased levels of physical activity were associated with reduced cancer risk, partly mediated through effects on body weight [22,23]. Notably, a recent study found that increased levels of the adipocyte-produced hormone leptin, that is typically elevated in obesity, and insulin resistance were associated with increased risk of oesophageal adenocarcinoma in men with Barrett’s oesophagus, independent of body mass index (BMI) and other known confounders [24].

Evidence from exercise trials in men and women at risk of either breast or colon cancer suggests that physical activity, at levels recommended for cancer prevention, can reduce adiposity, leptin, some inflammatory markers and insulin resistance [25–27]. Whether or not exercise can improve adiposity and obesity-associated hormones in males with Barrett’s oesophagus is unknown. Hence, current guidelines for the management of Barrett’s oesophagus do not provide any recommendations around lifestyle changes or weight loss [28,29]; most likely due to the lack of evidence on any beneficial or harmful effects.

The ‘Exercise and the Prevention of Oesophageal Cancer’ (EPOC) study aimed to investigate the effect of an exercise program versus stretching on risk factors associated with oesophageal adenocarcinoma development in overweight or obese, inactive males with Barrett’s oesophagus. The detailed trial protocol has been previously described [30]. It was hypothesized that serum concentrations of leptin, adiponectin (total and high molecular weight), interleukin-6 [IL-6], tumour necrosis factor-alpha [TNF-α], C-reactive protein [CRP], and insulin resistance [HOMA] would differ between participants randomized to the exercise group and the control group. The trial was designed to be powered to detect effects for these primary outcomes. Here we report on feasibility (recruitment rates, retention and intervention adherence), adverse events and provide an estimate of effect sizes on primary and secondary outcomes (waist circumference, body composition, fitness, strength and gastro-oesophageal reflux symptoms) to inform future trial development.

## Materials and Methods

The protocol for this trial and supporting CONSORT checklist are available as supporting information; see S1 CONSORT Checklist and S1 Protocol. EPOC was a two-arm, randomized controlled trial in males with Barrett’s oesophagus, conducted in Brisbane, Australia. Participants were recruited between May 2009 and September 2010, with data collected between May 2009 and March 2011. The trial was registered with the Australian New Zealand Clinical Trials Registry in June 2009 (ACTRN12609000401257; http://www.anzctr.org.au/). Due to the tight study timeline, participant recruitment opened prior to trial registration such that one participant had been recruited prior to the trial registration approval. There are no ongoing or related trials.

### Ethics Statement

The trial was approved by The University of Queensland Medical Research Ethics Committee (October 2008), Royal Brisbane and Women’s Hospital Human Research Ethics Committee (October 2008), UnitingCare Health Human Research Ethics Committee (April 2009), Princess Alexandra Hospital Human Research Ethics Committee (July 2009) and The Prince Charles Hospital Human Research Ethics Committee (December 2009). All participants provided written, signed informed consent.

### Participant Recruitment

Multiple strategies were used to recruit participants. Patients were primarily recruited through gastroenterology departments (three large tertiary hospitals, one private hospital and a private clinic) in Brisbane, Australia. Patients were provided with an information sheet by their treating doctor during routine appointments and given an expression of interest form to complete and post, or were identified from four hospital databases by nursing staff and mailed the information sheet and expression of interest form (n = 303). In addition, study information was mailed to Barrett’s oesophagus patients who had given permission to be included on a research study database (n = 100). Patients who returned the expression of interest form were contacted by study personnel via telephone to explain the study in further detail, screen for eligibility and answer any questions. Eligibility criteria included: Barrett’s oesophagus (defined as the abnormal appearance of the lining of the distal oesophagus on endoscopy, in addition to histological evidence of intestinal metaplasia on biopsy) [31]; male; age 18–70 years; and living in greater Brisbane, Australia (population 2 million). Exclusion criteria included: non-English speaking; body mass index (BMI) < 25.0 kg/m^2^ or > 34.9 kg/m^2^; performing > 60 min/week of moderate-vigorous intensity exercise during the previous 6-weeks; weight loss or gain ≥ 5kg during the past 6-months; and major co-morbidities (such as cardiac, respiratory, renal, liver, neurological or inflammatory disease). Patients with a BMI ≥ 35 kg/m^2^ were excluded due to increased risks of adverse events. Weight stability within ±5kg was included to ensure that the metabolic and inflammatory biomarkers of interest had been relatively stable prior to study commencement. Eligible participants who consented to study participation provided signed, written consent prior to baseline assessment. Following baseline data collection, participants were randomised to the exercise group or the control group. The randomization sequence was generated by a research assistant not involved in the study, using a computer-generated random number table (http://www.randomization.com), with block randomisation. Group allocation was concealed from the investigators using sealed, numbered envelopes. It is not possible to blind participants to the group allocation in exercise trials. However, all data were collected by research assistants blinded to group allocation and all primary outcomes were objectively measured, with laboratory staff blinded to study group.

### Exercise Intervention

The program consisted of 60 minute exercise sessions, five days/week for 24 weeks. Each session comprised 30 minutes of moderate-intensity (60–70% age predicted maximum heart rate) aerobic exercise and 30 minutes of resistance exercise (1–2 sets of 8–15 repetitions on major muscle groups) plus a 5-minute warm-up and cool-down. One session per week was performed at a hospital gymnasium in small groups under the supervision of study personnel (physiotherapist/exercise physiologist), the remaining four sessions were performed independently at a private health club (free health club memberships were provided). Participants performed aerobic exercise on treadmills, cycling or rowing stationary ergometers and elliptical machines. Exercise intensity was determined using the unmodified BORG scale [32]. Participants maintained their rate of perceived exertion between “somewhat hard” and “hard” [32]. Resistance exercises included: chest press, leg press, shoulder press, seated row, lunges, assisted chin up, assisted dip and core stability. Resistance was set at achieving muscular fatigue between 12–15 repetitions initially and then 8–10 repetitions after eight weeks of training. Resistance, therefore progressively increased as the subjects enhanced their strength. After 8 weeks, participants increased the number of sets completed from one to two. Adherence was monitored through daily participant-completed exercise diaries and attendance at the private health clubs was recorded electronically.

### Attention Control

Participants allocated to the control group attended a hospital gymnasium once a week to perform 45 minutes of stretching in small groups under the supervision of study personnel. They were also instructed to perform the stretching program independently at home, four days/week and not to commence a new exercise program during the study period. At the conclusion of the study participants in the control group were offered an exercise program and a complimentary three month membership to a private health club. Both exercise and control participants were instructed to not change their usual diet and to continue their regular anti-reflux medication regime for the duration of the trial.

### Data Collection

Demographic characteristics and medical history were obtained from an interviewer-administered questionnaire at baseline. All other measures were taken by trained blinded assessors at baseline, 12-weeks and 24-weeks except for medication use (documented daily), smoking habits (recorded every 4 weeks) and adverse events (documented in exercise diary). Participants recorded any adverse events (bodily complaint, injury or illness) in their daily exercise diaries regardless of the cause of the complaint. Exercise diaries were collected at the end of the study and any recorded adverse events were coded.

Physical activity was assessed using the International Physical Activity Questionnaire (IPAQ), (long form, last 7 days, self-administered format) [33,34]. Total energy intake was assessed using a validated, self-administered, 170-item food frequency questionnaire [35]. Body fat mass and lean mass were determined in triplicate by bioimpedance spectroscopy (ImpediMed SFB7, ImpediMed Ltd., Australia). Typical coefficients of variation within a measurement session range from 0.3 to 3.0% [36]. Waist circumference was measured in duplicate at the midpoint between the lower costal (rib) border and the iliac crest. Peak oxygen uptake (VO_2_peak) was quantified using a Cortex Metamax 3 portable metabolic analyser (Cortex: biophysik, GMbH, Germany) while participants performed the Modified Shuttle Walk Test [37]. One-repetition maximum (1RM) bench press and leg press tests measured muscle strength. Gastro-oesophageal reflux symptoms were measured using the Gastro-oesophageal Reflux Disease Impact Scale [38]. Fasting blood samples of 30 ml were collected by pathology personnel between 08:00–10:00am, at least 24 hours post exercise. Blood samples were centrifuged within 20 minutes of collection and stored at -80°C until assay.

### Blood Analysis

Serum leptin was analysed via radioimmunoassay (RIA) (Linco Research, Missouri, USA). The inter- and intra- coefficients of variation (CV) were < 8%. Serum adiponectin (total and HMW) was determined by an enzyme-linked immunosorbent assay (ELISA) (Alpco Diagnostics, Salem, USA). The inter- and intra-assay (CV) were < 8%. Serum concentrations of IL-6 and TNF-α were measured using a Milliplex suspension array system (Millipore, Billerica, USA), with inter-assay CV of < 8% and intra-assay CV of < 6%. Serum concentrations of CRP were analysed using a high sensitivity Particle Enhanced Immunonephelometry assay (Siemens Healthcare Diagnostics Products GmbH, Marburg, Germany). Inter- and intra-assay CV were ≤ 5%. Fasting serum insulin was analysed using an immunoenzymatic ‘sandwich’ assay via an ACCESS system (Beckman Coulter, Fullerton, USA) and plasma glucose concentrations determined with an oxygen rate method via the SYNCHRON system (Beckman Coulter, Fullerton, USA) with an inter- and intra-assay CV of < 5% for both insulin and glucose. Insulin resistance was calculated using the homeostasis model assessment (HOMA) formula (fasting insulin x fasting glucose/22.5) [39]. No biomarker values were under the laboratory’s detection limits. Participants were provided with a report of their blood results at the end of the study and were instructed to follow up any abnormal results with their doctor.

### Statistical Analysis

All data were entered into PASW statistical software (Version 18; SPSS inc., Chicago, IL). The analysis assessed the intervention effect (intervention—control) based on intention-to-treat principles. The amount of missing data was minimal at each assessment (n = 0 for blood, adiposity and GORD outcomes; n = 4 for fitness outcomes due to fault with measurement equipment; and n = 5 for strength outcomes due to musculoskeletal injury). In addition, two participants in the exercise group recorded extreme values for insulin and IL-6 at all assessments. Due to the small sample size, a decision was made post-hoc to exclude the data for all blood outcomes from these two participants to avoid undue influence for these outlying observations.

The mean changes in outcomes from baseline to week-24 were computed (mean changes at week-12 were included as supplementary analysis). Residuals of models for all outcomes, with the exception of IL-6 approximated a normal distribution. A univariate analysis of covariance (ANCOVA) assessed whether changes differed between the two groups at each follow-up assessment. Models were adjusted for baseline values of the outcome variable to account for any differences between groups at baseline and regression to the mean. Statistical tests were two-sided and p < 0.05 was considered statistically significant (as per trial protocol and sample size estimation). To account for testing multiple primary outcomes, p < 0.007 was considered statistically significant.

The study tested the null hypothesis that the mean difference between groups is zero and the alternative hypothesis that the mean difference is greater than zero. Between-group differences for mean changes in primary outcomes, that were greater than or equal to 10% (defined *a priori*), were considered noteworthy.

### Sample Size

We estimated a sample size of 40 participants per group (80 in total) was required to detect a minimum difference of 10% in primary outcomes between the exercise and control group, assuming 80% power, a type І error of 5% and allowing for 10% attrition (two-tailed) [30]. The 10% difference in means between groups and standard deviations for each of the outcomes were estimated from published data on each of the biomarkers in previous clinical trials in obese, inactive healthy males [40–47].

## Results

### Recruitment and Retention

**Fig 1:**
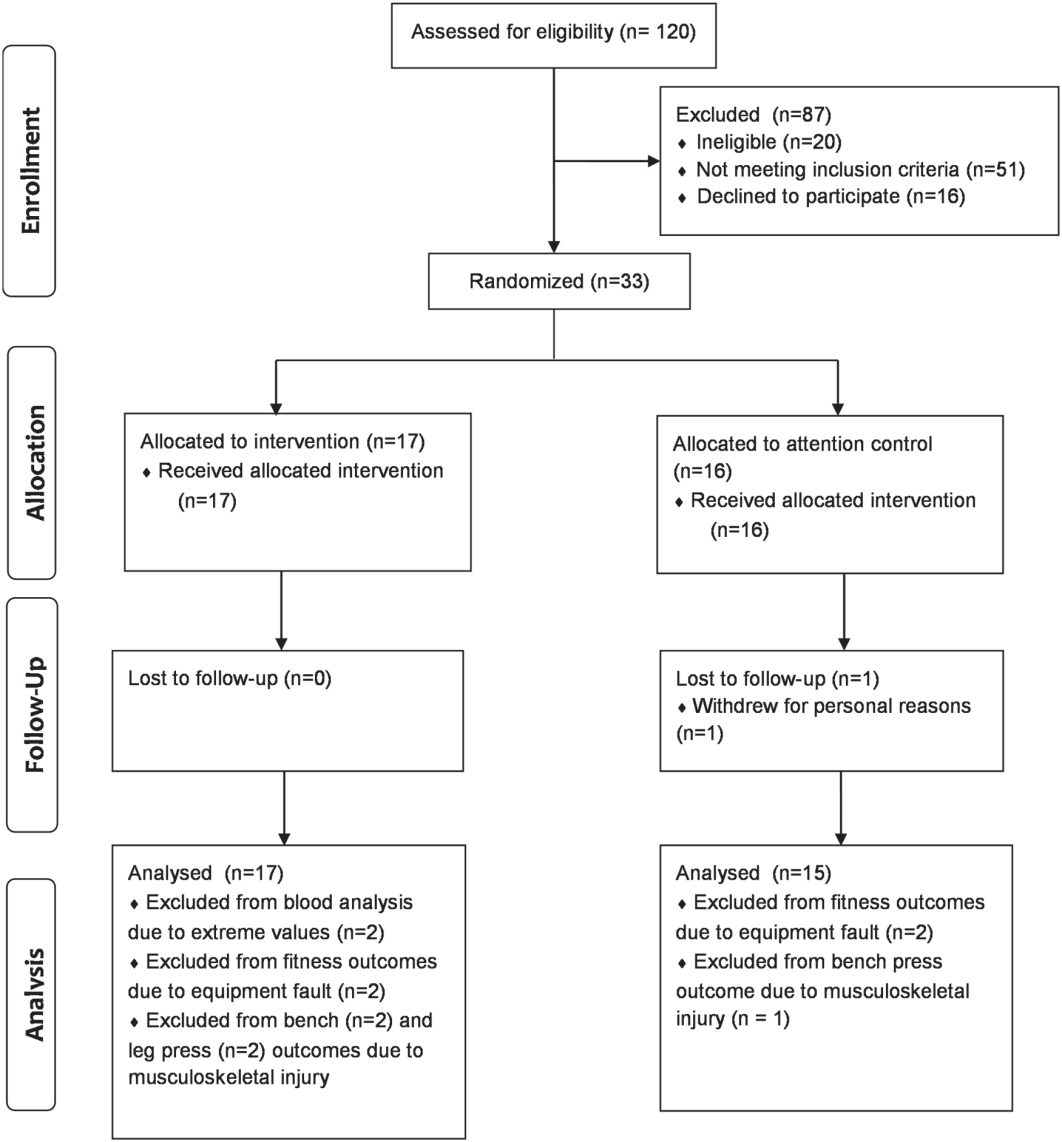
Participant flow diagram.

Of the 403 patients mailed study information, 113 (28.0%) expression of interest forms were received. Only seven expressions of interest forms were returned from patients who were provided the study information by their gastroenterologists during a routine consultation. Thus, a total of 120 men expressed interest in the study over the 17-month recruitment period (Fig. 1). Of these men, 20 (16.7%) did not meet the inclusion criteria (living outside study area [n = 11]; aged >70 years [n = 5]; not diagnosed with Barrett’s Oesophagus [n = 4]) and a further 51 (42.5%) were excluded. Reasons for exclusion included: participating in more than one hour/week exercise (n = 18); co-morbidity (n = 17); BMI <25.0 kg/m^2^ (n = 14); BMI >35.0 kg/m^2^ (n = 1); and >5kg weight loss in previous six months (n = 1). Of those eligible (n = 49), 16 (32.7%) men declined to participate (lack of time [n = 14]; no longer interested [n = 2]). A total of 33 participants provided informed consent and were randomly allocated to the exercise (n = 17) or control group (n = 16). Participants were primarily white, aged (mean ± SD) 57.4 ± 8.9 years and obese (30.1 ± 2.9 kg/m^2^). The majority of participants were taking anti-reflux medication (94%) and were classified as having ‘fairly well controlled’ reflux symptoms (55%), based on Gastro-oesophageal Reflux Disease Impact Scale scores. The two groups were similar in demographic characteristics, body composition, smoking, anti-reflux medication use, and reflux symptom control at baseline (Table 1). Despite randomization, mean baseline concentrations of leptin, adiponectin (total and HMW), and HOMA scores differed between the exercise and control groups by more than 10% (Table 2). Retention in the trial was excellent (97.0%) with only one participant withdrawing from the control group for personal reasons.

**Table 1:** Baseline characteristics of participants allocated to the exercise and control groups.

	Exercise Group	Control Group
n	17	16
Age (years)	57.2 ±7.5	57.6 ± 10.4
Body weight (kg)	94.7 ±10.5	92.4 ± 9.6
BMI (kg/m^2^)	30.8 ±2.6	29.4 ± 3.1
Peak oxygen consumption (ml/kg/min)	25.8 ± 8.2	26.8 ± 7.5
Moderate-to-vigorous exercise (min/week)^a^	0 (0, 43)	0 (0, 38)
Sitting time (h/wk)^a^	56 (35, 63)	41 (29, 52)
Total calories (kcal/day)	2252 ± 592	2176 ± 585
Smoking history		
Current smoker	1 (5.9)	1 (6.3)
Ex-smoker	8 (47.1)	5 (31.3)
Never-smoker	8 (47.1)	10 (62.5)
Anti-reflux medication		
Proton-pump inhibitor	15 (88.2)	14 (87.5)
H_2_-receptor antagonist	0 (0)	1 (6.3)
Over the counter	1 (5.9)	2 (12.5)
Nil	1 (5.9)	1 (6.3)
Gastro-oesophageal reflux symptoms^b^		
Very well controlled	2 (11.8)	5 (31.3)
Fairly well controlled	11 (64.7)	7 (43.8)
Uncontrolled	3 (17.6)	2 (12.5)
Poorly controlled	1 (5.9)	2 (12.5)
Highest level of education		
High school certificate	5 (29.4)	5 (31.3)
Technical college certificate	4 (23.5)	4 (25.0)
University degree	8 (47.1)	7 (43.8)
Employment		
Full-time	13 (76.5)	9 (56.3)
Part-time	0 (0)	3 (18.8)
Retired	4 (23.5)	4 (25.0)

Data are mean ± SD, median (25^th^, 75^th^ percentile) or n (%).

^a^ measured using International Physical Activity Questionnaire (recreational physical activity).

^b^ measured using Gastro-oesophageal reflux disease impact scale.

**Table 2:** Obesity-related hormone outcomes at baseline and week-24 comparing participants in the exercise group (n = 15) and control group (n = 15).

	Baseline	24-Weeks	Change from baseline to 24-weeks		Intervention effect (Exercise—Control)	
	Mean (SD)	Mean (SD)	Mean (95%CI)	% change	Mean (95% CI)	p-value ^a^
Leptin (ng/mL)						
Exercise group	11.7 (7.7)	9. 4 (6.5)	-2.4 (-5.0, 0.08)	-20.5	-3.0 (-6.6, 0.6)	0.09
Control group	13.0 (9.2)	13.6 (10.7)	0.6 (-1.9, 3.1)	4.6		
Total adiponectin (μg/mL)						
Exercise group	6.5 (2.7)	5.1 (1.8)	-1.3 (-1.8, -0.7)	-16.9	0.26 (-0.5, 1.0)	0.48
Control group	5.9 (2.8)	4.5 (1.5)	-1.6 (-2.3, -0.8)	-27.8		
HMW adiponectin (μg/mL)						
Exercise group	2.8 (1.5)	2.0 (1.8)	-0.6 (-1.0, 0.2)	-17.8	-0.06 (-0.6, 0.5)	0.83
Control group	2.1 (1.4)	1.7 (1.0)	-0.5 (-1.0, 0.1)	-23.8		
IL-6 (pg/mL)						
Exercise group	4.3 (3.4)	4.5 (4.7)	-	-	0.93 (0.56, 1.55)^b^	0.77
Control group	4.3 (5.2)	4.5 (4.5)	-	-		
TNF-α (pg/mL)						
Exercise group	7.0 (2.8)	7.3 (3.5)	0.3 (-1.1, 1.7)	4.3	0.34 (-1.7, 2.3)	0.73
Control group	6.5 (1.9)	6.5 (3.1)	-0.01 (-1.4, 1.4)	-0.2		
CRP (mg/L)						
Exercise group	3.5 (6.3)	1.9 (1.7)	-1.6 (-2.4, -0.7)	-45.7	0.61 (-0.6, 1.8)	0.32
Control group	3.5 (5.1)	1.3 (1.5)	-2.1 (-2.9, -1.2)	-60.0		
HOMA						
Exercise group	2.6 (1.1)	2.2 (1.1)	-0.47 (-0.9, 0.0)	-18.2	-0.05 (-0.7, 0.6)	0.88
Control group	3.6 (2.3)	3.2 (2.5)	-0.43 (-0.9, 0.0)	-11.9		

HMW, high molecular weight; IL-6, interleukin-6; TNF-α, tumour necrosis factor-alpha; CRP, C-reactive protein; HOMA, homeostasis model assessment.

^a^ Change in exercise group versus change in control group, adjusted for baseline value (ANCOVA).

^b^ Backtransformed from natural log; expressed as relative ratio.

### Adherence

Based on all exercise (supervised and unsupervised) recorded in the daily exercise diaries over the 24-week study period, participants in the exercise group recorded a median of 3.5 hours of exercise per week (25^th^, 75^th^ percentiles: 3.1, 5.3 hrs/week). Exercise group participants attended 78% of their supervised sessions and 56% of their independent gym-based sessions based on daily exercise diaries (53% attendance based on electronic records) and recorded a median of 0.5 hours (0.1, 1.3 hours) of non-protocol exercise per week (e.g. mountain biking, swimming). Control group participants recorded a median of 0.2 hours: 0.0, 0.9 hours) of non-protocol exercise per week on their daily exercise diaries and attended 56% of their supervised stretching sessions. Although control group participants were advised to not change their usual physical activity levels, five participants (31%) recorded greater than one hour of exercise per week on at least 5 weeks over the study period. Compared to control group participants, participants in the exercise groups self-reported significantly more leisure-time physical activity, but no difference in occupational and household physical activity levels and sitting time (S1 Table). There was a significant difference in change in total energy intake between groups from baseline to 24-weeks (p = 0.04; S1 Table).

### Adverse Events

Adverse events attributable to the study included minor musculoskeletal complaints (n = 4), which was similar between the exercise (n = 2) and control (n = 2) groups. No severe adverse events were reported. Mild muscle stiffness/soreness was reported by 11 (64.7%) intervention participants.

### Primary Outcomes

Changes in primary outcomes from baseline to 24-weeks are shown in Table 2 (changes from baseline to 12-weeks are shown in S2 Table; medians [25^th^, 75^th^ percentiles] at baseline, 12-weeks and 24-weeks are shown in S3 Table). There were no statistically significant between-group differences for changes in any of the obesity-associated hormones measured (Table 2).

### Secondary Outcomes

There were no statistically significant between-group differences in body weight and body composition at 24-weeks (Table 3), although a statistically significant intervention effect on waist circumference was observed (-4.5 [95% CI-7.5, -1.4] cm; p < 0.01). There was no significant difference in gastro-oesophageal reflux symptoms between groups at 24-weeks. Compared to control group participants, participants in the exercise groups observed greater improvements in cardiovascular fitness and upper-body strength but not lower-body strength. Changes from baseline to 12-weeks are shown in S4 Table.

**Table 3:** Body composition, fitness, strength and gastro-oesophageal reflux outcomes at baseline and week-24 comparing participants in the exercise group and control group.

		Baseline	24-Weeks	Change from baseline to 24-weeks		Intervention effect (Exercise—Control)	
	n	Mean (SD)	Mean (SD)	Mean (95%CI)	% change	Mean (95%CI)	p-value^a^
Waist circumference (cm)							
Exercise group	17	105.3 (8.2)	100.2 (4.9)	-5.1 (-7.1, -2.9)	-4.8	-4.4 (-7.5,-1.4)	< 0.01
Control group	15	104.8 (10.6)	104.2 (9.7)	-0.6 (-2.9, 1.6)	-0.6		
Weight (kg)							
Exercise group	17	94.7 (10.5)	92.3 (12.1)	-2.5 (-4.1, -0.8)	-2.6	-2.0 (-4.4,0.4)	0.10
Control group	15	92.4 (9.6)	91.5 (9.5)	-0.5 (-2.2, 1.3)	-0.5		
Fat mass (kg)							
Exercise group	17	22.0 (6.1)	19.9 (6.5)	-2.0 (-4.2, 0.2)	-9.2	-1.6 (-4.9,1.7)	0.32
Control group	15	21.6 (8.7)	21.4 (6.2)	-0.3 (-2.7, 2.0)	-1.5		
Lean Mass (kg)							
Exercise group	17	72.6 (10.3)	72.3 (10.4)	-0.1 (-2.5, 2.3)	-0.1	0.4 (-3.1,4.0)	0.80
Control group	15	70.9 (7.6)	70.1 (6.9)	-0.5 (-3.1, 2.0)	-0.7		
VO_2_ peak (mL/min/kg)							
Exercise group	15	26.1 (8.2)	32.2 (7.1)	5.5 (2.2, 8.7)	21.1	4.5 (-0.3,9.2)	0.06
Control group	13	26.8 (7.5)	27.9 (5.8)	1.0 (-2.4, 4.4)	3.7		
Bench press (kg)							
Exercise group	15	37.1 (14.3)	47.0 (13.9)	11.7 (7.1, 16.4)	31.5	9.7 (3.0,16.3)	< 0.01
Control group	14	35.0 (13.3)	36.8 (14.1)	2.1 (-2.7, 6.8)	6.0		
Leg Press (kg)							
Exercise group	15	136.5 (35.2)	167.8 (35.7)	32.6 (17.8, 47.3)	23.9	11.7 (-9.5,33.0)	0.26
Control group	15	140.2 (46.6)	161.5 (48.1)	20.8 (5.6, 36.1)	14.8		
Gastro-oesophageal reflux^b^							
Exercise group	17	10.8 (1.0)	11.4 (0.7)	0.5 (0.0, 1.0)	4.6	0.26 (-0.5,1.0)	0.46
Control group	15	10.8 (1.5)	11.2 (1.2)	0.2 (-0.3, 0.7)	1.9		

^a^ Change in exercise group versus change in control group, adjusted for baseline value (ANCOVA).

^b^ Measured using Gastro-oesophageal reflux disease impact scale.

## Discussion

This is the first trial to attempt an exercise intervention in men with Barrett’s oesophagus and provides useful pilot data to inform effect size estimation in future larger-scale trials. Overall, recruitment was difficult in this population and the target sample size of 80 participants was not achieved. Although no statistically significant between-group differences were observed for any of the primary outcomes there was some evidence to suggest that exercise may have potentially meaningful effect on leptin.

Despite recruitment via a number of sources, the relatively low prevalence of Barrett’s oesophagus in the population (variously estimated at approximately 1–2%) [6,7] and the strict eligibility criteria employed in this study meant that we did not reach our sample size target. The study was therefore underpowered to detect differences in all of the outcomes associated with oesophageal cancer risk (for example, the sample provided less than 70% power to detect the difference observed in leptin concentrations and total body weight and less than 30% power to detect the difference observed in fat mass). Consequently findings of this trial must be considered exploratory but provide useful pilot data to estimate effect sizes for future trials.

Due to the lack of evidence on exercise intervention in Barrett’s oesophagus patients, this pilot study was designed as an efficacy trial (versus an effectiveness trial) and aimed to recruit a relatively homogenous sample of Barrett’s oesophagus patients with a strict intervention protocol including attendance at supervised exercise sessions [48]. Approximately half of the men, who expressed interest in the study and met inclusion criteria, were excluded due to the strict eligibility criteria. In addition, almost a third of eligible patients declined to participate in the study, primarily due to lack of time. Low levels of recruitment and participation may be a recurring challenge for intervention research in Barrett’s oesophagus. By analogy, a dietary and weight loss trial targeting a very similar patient population in the US with biomarker endpoints had a similar rate of recruitment as this study [12]. Due to the minimal adverse events reported, future trials should consider using less strict eligibility criteria and/or recruiting across multiple cities. Direct mailing of study information sheets to patients identified from hospital and research databases, was the most effective recruitment strategy in this study. Use of a dedicated clinical trials/research nurse to identify and recruit patients in clinics, rather than relying on clinicians to provide study information to patients during routine appointments, would also assist with recruitment [49]. Subsequent effectiveness-based trials which used different or more flexible strategies for increasing exercise participation, may assist to increase the reach and representativeness of those recruited into the trial.

The exercise protocol included one supervised and four unsupervised sessions (with free gym membership provided). While adherence to the single supervised session was good (78%), adherence to the four independent gym-based exercise sessions was moderate (56%). Previous exercise trials for primary prevention of cancer have included 2–3 supervised sessions per week with the unsupervised sessions primarily as home-based exercise [25]. A greater number of supervised exercise sessions may have improved adherence to the exercise protocol in the current study. Furthermore, almost a third of participants in the attention control group reported exercising for more than one hour per week on at least five weeks over the study period; thus the intervention effects observed on the primary outcomes may be underestimated. To minimise contamination, stretching sessions were held at different times to the exercise group sessions and attention control group participants were instructed to maintain their usual physical activity levels. However contamination is common in exercise trials, where participants can freely choose to engage in the ‘active treatment’, as opposed to placebo-controlled drug trials. Contamination rates in control groups in other exercise trials have ranged from 12% to 52% [50,51], which serve to under-estimate the treatment effect.

In this trial, a statistically significant reduction in waist circumference was observed after 24-weeks of exercise, compared to attention-control stretching. Visceral adiposity has recently been shown to be a more important risk factor for oesophageal adenocarcinoma than measures of overall obesity (such as BMI) [18–20]. The reduction in waist circumference following the exercise intervention may therefore confer an important reduction in cancer risk for these men with Barrett’s oesophagus. Findings from a large cohort study indicated a 16% (95% CI 1.04–1.29) increased risk of oesophageal adenocarcinoma for every 5.0cm increase in waist circumference [18]. Future trials should consider inclusion of more accurate measures of body composition, including body fat distribution [19].

No statistically significant effect on any of the primary outcomes was observed. While it is possible that the exercise had no beneficial effect on these biomarkers, low power (inadequate sample size) must also be considered, as 95% confidence intervals were wide. Leptin, in particular, for which a non-significant but clinically meaningful effect (>10% difference defined *a priori*) was observed in this sample (-3.0 [95% CI-6.6, 0.6] ng/mL; p = 0.09), may warrant further investigation in larger studies, considering the substantial evidentiary support from the literature for the potential relevance of this biomarker. Leptin, a hormone secreted by adipocytes and increased in the obese state, has been postulated in the development of oesophageal adenocarcinoma as well as other cancers [52,53]. *In vitro*, leptin has been shown to elicit mitogenic, angiogenic and anti-apoptotic effects when administered to oesophageal adenocarcinoma cell lines, enhancing cellular proliferation [54–57]. Recently, elevated leptin concentrations have been associated with progression from Barrett’s oesophagus to oesophageal adenocarcinoma in men independent of adiposity [24]. Similarly, increased leptin levels have been shown to be associated with increased risk of Barrett’s oesophagus in males, independent of adiposity [41,58]. The reduction in leptin concentrations following the exercise intervention in the present study (-21.2% in exercise group vs. +4.6% in control group) is therefore likely to be important in reducing risk of oesophageal adenocarcinoma. Intervention trials in inactive, overweight adults without Barrett’s oesophagus have also observed significant reductions in leptin following exercise, with these reductions largely mediated by reductions in adiposity [26,59].

No significant intervention effect was observed for adiponectin, insulin resistance, or inflammatory markers (CRP, IL-6 and TNF-a) although the findings were inconclusive. Concentrations of CRP, HOMA and total adiponectin however significantly reduced within both groups from baseline to week-24. While the stretching group was included to control for contact between study groups, even this minimal-intensity program (one supervised stretching session/week and four independent sessions/week; 45 mins/session) may have led to improvements in some hormonal and inflammatory markers via a reduction in psychological stress [60,61] or metabolic mechanisms [62]. More substantial reductions in adiposity may be necessary to increase adiponectin [63] (a hormone which usually increases with decreasing adiposity [64] and reduce inflammatory markers such as IL-6 and TNF-a [65]. Results of exercise interventions in populations without Barrett’s oesophagus have shown conflicting effects on changes in adiponectin concentrations [64,66]. The reduction in adiponectin concentration observed (despite modest weight loss) may be due to concomitant alterations in other hormones and cytokines in response to exercise alone [64,67,68]. The magnitude of weight loss observed in the EPOC trial is consistent with that observed in other exercise only interventions [69,70]. Interventions targeting both diet and exercise are necessary to achieve greater weight loss [71,72], and may be more likely to result in beneficial changes in adipokines and inflammatory markers [64].

The only other study to examine a lifestyle intervention in adults with Barrett’s oesophagus to date focused on a dietary-only intervention (low fat, high fruits and vegetables) and weight loss [12]. A between-group difference in weight of-4.0kg was observed at 18-months but no significant effects on markers of cellular proliferation, re-epithelialization or Barrett segment length were observed. Findings from the study by Kristal and colleagues [12] and our study, suggest that greater weight loss may be necessary to substantially reduce risk of oesophageal adenocarcinoma in men with Barrett’s oesophagus.

The primary limitation of the study is the small sample size and low statistical power, as previously noted. Additionally, the use of biomarkers associated with oesophageal adenocarcinoma risk as outcomes cannot determine whether a causal relationship exists between physical activity and oesophageal cancer in men with Barrett’s oesophagus; such a trial would not be feasible to conduct. Measuring associated biomarkers can however, provide important information about the possible benefits and harms of physical activity interventions in individuals with Barrett’s oesophagus, in addition to increasing the understanding of the underlying biologic pathways potentially involved in exercise and cancer prevention. Furthermore, assessment of unsupervised exercise sessions was based on self-reported exercise diaries. Objective measurement of physical activity via accelerometers or other wearable devices that measure both duration and intensity over longer periods, in future trials would assist in assessing adherence to the intervention and monitoring cross-contamination. More frequent assessments of actual dietary intake, for example with a daily food diary, would provide better assessment of changes in dietary intake throughout the study period.

## Conclusion

This small, exploratory trial provides novel evidence that a moderate-intensity aerobic and resistance exercise intervention is feasible to deliver in overweight and inactive men with Barrett’s oesophagus and resulted in modest beneficial effects on waist circumference and leptin concentrations, important risk factors for oesophageal adenocarcinoma. It is likely that greater changes in adiposity, in particular reductions in visceral adiposity, may be required to substantially reduce cancer risk in men with Barrett’s oesophagus. Future research should investigate a combined dietary and exercise intervention on these intermediate biomarkers in this at risk and understudied population. Such evidence would inform recommendations around lifestyle changes for adults with Barrett’s oesophagus.

## Supporting Information

S1 CONSORT Checklist (PDF)

S1 File Ethics Approval Letter. (PDF)

S2 File Information Sheet and Consent Form. (PDF)

S3 File Data Record Form. (PDF)

S1 Protocol Trial Protocol. (PDF)

S1 Table Physical activity* and dietary outcomes at baseline and week-24 comparing participants in the exercise group (n = 16) and control group (n = 15). (DOCX)

S2 Table Obesity-related hormone outcomes at baseline and week-12 comparing participants in the exercise group (n = 15) and control group (n = 16). (DOCX)

S3 Table Median (25th, 75th percentile) of obesity-related hormones at baseline, week 12 and week 24. (DOCX)

S4 Table Body composition, fitness, strength and gastro-oesophageal reflux outcomes at baseline and week-12 comparing participants in the exercise group and control group. (DOCX)
