# Supplementary material for: The Feasibility of an Exercise Intervention in Males at Risk of Oesophageal Adenocarcinoma: A Randomized Controlled Trial
Source: PLoS One. 2015 Feb 23;10(2):e0117922. doi: 10.1371/journal.pone.0117922 (PMC4338269; doi:10.1371/journal.pone.0117922)
Supplement: S1 File — (PDF) [file pone.0117922.s002.pdf]

Royal Brisbane and Women's Hospital
Health Service District

Queensland Government

Queensland Health

Office of the Human Research Ethics Committees

Enquiries to: Odette Petersen Coordinator
Phone: 07 3636 5490
Fax: 07 3636 5849
Our Ref: 2008/110
E-mail RBWH-Ethics@health.qld.gov.au

Ms Brooke Winzer
Burns, Trauma & Critical Care Research Centre
UQ Centre for Clinical Research
Level 8, Building 71/918
Royal Brisbane & Women's Hospital
HERSTON Q 4029

Dear Ms Winzer,

Re: Reference No. 2008/110: Can We Alter Risk Factors for Oesophageal Cancer with Exercise?

At a meeting of the Royal Brisbane & Women's Hospital Human Research Ethics Committee held on 8 September, 2008, the Committee reviewed the above protocol. The Royal Brisbane & Women's Hospital Human Research Ethics Committee is duly constituted, and operates and complies with the National Health and Medical Research Council's *"National Statement on Ethical Conduct in Human Research 2007"*. The Chairperson of the HREC reviewed your further correspondence on 6 October, 2008.

It is advised that on the recommendation of the Human Research Ethics Committee, the Clinical Chief Executive Officer, Royal Brisbane & Women's Hospital has approved your request for ethical approval of the following:

1. Letter of Support from Director, Burns, Trauma & Critical Care Research Centre, Department of Intensive Care Medicine, Royal Brisbane & Women's Hospital dated 08/08/08
2. NEAF Version 1.1 dated 05/08/08
3. Attachment A – 1.2.1 Description of the Project in Plain Language
4. Attachment B – 3.2.1 Description of the Theoretical, Empirical and Conceptual Basis, and Background Evidence, for the Research Proposal
5. Attachment C – 3.2.3 Research Design and Methods
6. Attachment D – 4.1.6.5 Endpoints
7. Attachment E – 5.1.3.5 Participant Characteristics and how they are Relevant to the Aims of the Study
8. Attachment F – 5.2.1 What Participants will Experience
9. Attachment G – Recruitment Process Flowchart

Office	Postal	Phone	Fax
Herston Rd<br>Herston Q 4029	Post Office Herston<br>Queensland 4029 Australia	07 3636 5490<br>ISD + 61 7 3636 5490	07 3636 5849

10. Attachment H – 5.4.4.1.1 Phone Call Script
11. Participant Information and Consent Form, Version 2 dated 26/09/08
12. Expression of Interest Form, Version 1 dated 26/07/08
13. Primary Contact Practitioner Information Form, Version 1 dated 26/07/08
14. Inclusion & Exclusion Criteria Table
15. Body Mass Index (BMI) for Adults Chart
16. The Barrett's Exercise Study Recruitment Poster
17. Demographic Questionnaire
18. Gastroesophageal Reflux Questionnaire (GERQ) – Developed by the Mayo Clinic 1993
19. Food Frequency Questionnaire – Developed by QIMR 2003
20. Medication Log
21. Barrett's Exercise Study Diary
22. Finance Statement

During the conduct of the study you are required to adhere to the following conditions:

- All research projects undertaken with the branches of CaSS (either by CaSS staff or by researchers using CaSS resources) must gain approval to proceed from the Executive Director via the CaSS Research Committee.

- If recruitment has not commenced within 12 months, please advise the Coordinator, HREC.

- All forms required when submitting reports to the HREC are accessible on the Herston Intranet and Internet. In the first instance please access the Commencement Form and return to this office when the study commences. Please contact the Coordinator if you do not have access to this site.

- In accordance with RBWH Policy 72005: Clinical Trial Documentation, all medical records of research participants must contain documentation regarding the patient's involvement in the trial.

- All investigations must be carried out according to the "Declaration of Helsinki 2004" as subsequently modified and the latest statement by the National Health and Medical Research Council on Human Experiments and on Scientific Practice. Should a copy of the 'Declaration of Helsinki 2000' as subsequently modified be required, please request a copy from the Coordinator, Human Research Ethics Committee.

- Attachment I is a letter listing some matters specified by the National Health and Medical Research Council to which you as the research worker must adhere.

- Attachment II gives the Committee composition with specialty and affiliation with the Royal Brisbane & Women's Hospital.

- You are required to provide a report on any pilot study and the outcome of the study at the completion of the trial or **annually** if the trial continues for more than 12 months.

- If any subsequent change/amendment is made to the protocol it will be necessary for you to obtain approval from the Human Research Ethics Committee. In addition a summary of the

amendments and a comment is required from the Principal Investigator. All amended documents must contain revised version numbers, version dates and page numbers. Changes must be highlighted using Microsoft Word "Track Changes" or similar. Please contact the HREC Coordinator if assistance is required.

- Serious Adverse Events must be notified to the Committee as soon as possible. In addition the Investigator must provide a summary of the adverse events, in the specified format, including a comment as to suspected causality and whether changes are required to the Patient Information and Consent Form.

- If the results of your protocol are to be published, an appropriate acknowledgment of the Hospital should be contained in the article. Copies of all publications resulting from the study should be submitted to the Human Research Ethics Committee.

- Please ensure that a copy of any publication that results from this protocol is also forwarded to the Herston Medical Library for future reference.

- The Hospital administration and the Human Research Ethics Committee may inquire into the conduct of any research or purported research, whether approved or not and regardless of the source of funding, being conducted on hospital premises or claiming any association with the Hospital; or which the Committee has approved if conducted outside the Royal Brisbane & Women's Hospital Health Service District. This may include consultation with the Principal Investigator and/or a visit to the research site by a member of the HREC and/or Coordinator of the HREC.

Should you have any problems, please liaise directly with the Chairperson of the Human Research Ethics Committee early in your program.

We wish you every success in undertaking this research.

Yours faithfully,

Dr Conor Brophy
Chairperson
RBWH Human Research Ethics Committee
10/10/2008
