# Supplementary material for: The Feasibility of an Exercise Intervention in Males at Risk of Oesophageal Adenocarcinoma: A Randomized Controlled Trial
Source: PLoS One. 2015 Feb 23;10(2):e0117922. doi: 10.1371/journal.pone.0117922 (PMC4338269; doi:10.1371/journal.pone.0117922)
Supplement: S2 File — (PDF) [file pone.0117922.s003.pdf]

Participant Information and Consent Form

Version 2 Dated 26/09/08
Site: Royal Brisbane & Women's Hospital (RBWH)

Full project Title: Can we Alter Risk Factors for Oesophageal Cancer with Exercise?

Principal Researchers: Ms Brooke Winzer (PhD student, Burns, Trauma and Critical Care Research Centre), Dr Jennifer Paratz (Chair of the Burns, Trauma and Critical Care Research Centre), Associate Professor David Whiteman (School of Public Health, Dept of Epidemiology, Queensland Institute of Medical Research) & Dr Mark Appleyard (Director of Endoscopy, Gastroenterological Services, Royal Brisbane & Women's Hospital)

Associate Researcher: Ms Kellie Stockton (Research Assistant, Burns, Trauma and Critical Care Research Centre)

This Participant Information and Consent Form is **8** pages long. Please make sure you have all the pages.

---

1. Your Consent

You are invited to take part in this research project.

This Participant Information form contains detailed information about the research project. Its purpose is to explain to you as openly and clearly as possible all the procedures involved in this project before you decide whether or not to take part in it.

Please read this Participant Information carefully. Feel free to ask questions about any information in the document. You may also wish to discuss the project with a relative or friend or your local health worker. Feel free to do this.

Once you understand what the project is about and if you agree to take part in it, you will be asked to sign the Consent Form. By signing the Consent Form, you indicate that you understand the information and that you give your consent to participate in the research project.

You will be given a copy of the Participant Information and Consent Form to keep as a record.

2. Purpose and Background

Barrett's oesophagus (BE) is an acquired condition in which the cells lining the oesophagus (or "gullet") transform into cells resembling the lining of the intestines. The condition is a risk factor for developing cancer of the oesophagus. It is estimated that 0.2 - 2% of people with BE will progress to oesophageal cancer per year. Other risk factors for developing cancer of the oesophagus include: male gender, being overweight and experiencing reflux (heart burn). Altered levels of hormones in the blood may also increase cancer risk. It is not known whether exercise can change body weight or hormone levels among people with BE which in turn may alter their risk of developing cancer.

This is the first study to investigate the effect exercise has on some of the risk factors associated with cancer of the oesophagus.

A total of 120 overweight men with Barrett's oesophagus will participate in this project. The project will be 6 months in duration.

If you agree to be in this study, you will perform an exercise program 5 days per week for 6 months. To evaluate the effect of the exercise program on risk factors for oesophageal cancer, a number of tests will be performed on 3 occasions.

3. Procedures

Participation in this study requires you to

- Provide **demographic information** e.g. age, gender, occupation, education level, smoking history, alcohol consumption, physical activity levels and diet during the previous 6 months, medication use, date of Barrett's Oesophagus diagnosis, previous endoscopy results, and details of any other medical conditions.

- Attend the RBWH to complete the following measurements 3 times over a 6 month period
    - **Modified Shuttle Walk Test.** You will be asked to walk along a 10 meter course that is marked by 2 cones. Walking speed is set by an audio signal on a CD. The initial speed is very slow but increases every minute. The test ends when you feel that you cannot go any further or you cannot keep up with the speed of the CD. This test is repeated after a rest of at least thirty minutes as people usually perform better on the second test. During the second test you will wear a facemask that is connected to a computer system attached to a light harness worn around your shoulders. This measures the amount of

oxygen you use during the test which is a measure of fitness. This is not distressing or painful.

- **One-repetition maximum strength test**. This test requires you to lift a particular weight once to see if it is the heaviest weight you can lift only one time. This is a quick and simple method of determining muscular strength.
- **Blood test.** A 30ml fasting blood sample will be taken by a research nurse from a vein in your arm.
- **Height, waist and hip circumference** will be measured with a tape measure.
- **Body weight** will be determined by standing on a scale.
- **Body fat** will be measured with a bio-impedance analyser (BIA). This machine uses a probe placed gently on the abdomen. The BIA relies on passing a small electric current through the body and measuring impedance (resistance) to the current. This is dependent on total body water, and in combination with information on weight, height and gender, allows estimation of body fat percentage and fat mass. It is not distressing or painful.

- Complete 2 **questionnaires** regarding reflux (heart burn) symptoms and about your diet.

- Complete a **physical activity diary** and a **medication log** on a daily basis. You will be asked to write down how much physical activity you performed each day and note the drug name, dose and time taken of any medications you use.

- Complete one of two different **exercise programs** for 6 months. As we do not know the effect exercise has on people with Barrett's oesophagus we will randomly divide participants into two different exercise groups. Half of the people in this study will be provided with a low intensity exercise program and half will be given a moderate intensity exercise program.

  a. **Low Intensity Exercise Program**: Participants will perform whole body stretching. This program will be performed under the supervision of a physiotherapist at the RBWH once a week. Participants will also be required to perform the program independently at home four times per week. Exercise sessions will be between 30-60 minutes in duration.
  b. **Moderate Intensity Exercise Program**: Participants will perform weight training and use fitness equipment such as treadmills, exercise bikes and rowing machines. One session per week will be completed at the RBWH under the direction of a physiotherapist. Four exercise sessions will be performed at a local gymnasium independently. Exercise sessions will be 60 minutes in duration.

4. Possible Benefits
At the end of the study all participants will be given information regarding which exercise program reduced oesophageal cancer risk factors the most. However, it is not known if exercise can benefit people with Barrett's oesophagus at all, as no other studies have been performed in this area previously. Also, any likely benefits may require continued exercise participation to be achieved.

5. Possible Risks
Occasional side effects of fitness testing include problems with heart rhythm, angina or heart attacks, low blood pressure, fainting, asthma attacks and very rarely death. Research studies of patients undergoing similar testing for heart problems have reported the risk of death being less than 1 in 10 000 tests. A physiotherapist is present throughout the Modified Shuttle Walk Test and the test can be terminated by you at any time. The physiotherapist will terminate the test if he/she determines that it is unsafe for you to continue exercising.

Injuries to muscles, tendons or ligaments can occur with exercise. To minimize this risk all exercise programs will be tailored to each individual's level of ability and gradually progressed by an experienced physiotherapist.

With any blood test there is a slight risk of excessive bleeding, infection, hematoma (blood collecting under the skin) and fainting.

6. Alternatives to Participation
Alternative procedures involve not participating in this project. You will continue to receive routine care for the management of Barrett's oesophagus. Not participating in this project will not affect your care in any way.

7. Privacy, Confidentiality and Disclosure of Information
The figures we record from you e.g. blood and exercise test results will be stored under a study number not an actual name. The data will be coded and can be potentially identifiable. Any form that can identify you e.g. consent form will be stored separately to the other information and will be kept for 15 years in accordance with hospital policy. It will then be destroyed in a confidential shredder. Only the investigators in this study can access this information.

At the conclusion of the study all blood samples will be destroyed. You may choose to withdraw your blood sample from the study at any time.

Any information that can identify you will remain confidential. It will only be disclosed with your permission, except as required by law. If you give us your permission by signing the Consent Form, we plan to publish the results in medical journals and present the information at conferences.

In any publication, a study number will be used and the information will be presented as group data i.e. an average of figures obtained rather than individual patient results.

8. New Information Arising During the Project
During the research project, new information about the risks and benefits of the project may become known to the researchers. If this occurs, you will be told about this new information.

9. Results of Project
When the project is finished we will summarize the information in a short report. The report will be posted to you on request.

10. Further Information or Any Problems
If you require further information or if you have any problems concerning this project (for example, any side effects), you can contact a principal researcher. The researchers responsible for this project are:

Ms Brooke Winzer – Principal researcher (07) 33466081
Dr Jennifer Paratz – Principal researcher (07) 3636 4113

This study has been reviewed and approved by the Royal Brisbane & Women's Hospital Health Service District Human Research Ethics Committee. Should you wish to discuss the study with someone not directly involved, in particular in relation to matters concerning policies, information about the conduct of the study or your rights as a participant, or should you wish to make an independent complaint, you can contact the Coordinator or Chairperson, Human Research Ethics Committee, Royal Brisbane & Women's Hospital, Herston, QLD 4029 or telephone (07) 3636 5490 or email RBWH-Ethics@health.qld.gov.au

This study adheres to the Guidelines of the ethical review process of The University of Queensland. Whilst you are free to discuss your participation in this study with project staff (contactable on 07 3636 4113), if you would like to speak to an officer of the University not involved in the study, you may contact the Ethics Officer on 3636 3924.

11. Other Issues
If you have any complaints about any aspect of the project, the way it is being conducted or any questions about your rights as a research participant, then you may contact

Name: The Co-ordinator
Position: Administrator Human Research Ethics Committee, RB&WH
Telephone: 07 3636 5490

You will need to tell the co-ordinator the name of one of the researchers given in section 10 above.

12. Participation is Voluntary

Participation in any research project is voluntary. If you do not wish to take part you are not obliged to. If you decide to take part and later change your mind, you are free to withdraw from the project at any stage.

Your decision whether to take part or not to take part, or to take part and then withdraw, will not affect your relationship with the Royal Brisbane & Women's Hospital.

Before you make your decision, a member of the research team will be available to answer any questions you have about the research project. You can ask for any information pertaining to the study that you want. Sign the Consent Form only after you have had a chance to ask your questions and have received satisfactory answers.

If you decide to withdraw from this project, please notify a member of the research team before you withdraw.

13. Ethical Guidelines

This project will be carried out according to the *National Statement on Ethical Conduct in Research Involving Humans* (March 2007) produced by the National Health and Medical Research Council of Australia. This statement has been developed to protect the interests of people who agree to participate in human research studies.

The ethical aspects of this research project have been approved by the Human Research Ethics Committee of Royal Brisbane & Women's Hospital, the University of Queensland and the Queensland Institute of Medical Research.

14. Reimbursement for your costs

You will not be paid for your participation in this project. Parking vouchers for the Royal Brisbane & Women's Hospital car park will be provided. Participants allocated the moderate intensity exercise program will be provided with a 6 month gym membership the Fitness First gym of their choice.

Consent Form
Version 2 **Dated** 26/09/08
Site: Royal Brisbane & Women's Hospital

Full project Title: Can we Alter Risk Factors for Oesophageal Cancer with Exercise?

Principal Researchers: Ms Brooke Winzer (PhD student, Burns, Trauma and Critical Care Research Centre), Dr Jennifer Paratz (Chair, Burns, Trauma and Critical Care Research Centre), Associate Professor David Whiteman (School of Public Health, Dept of Epidemiology, Queensland Institute of Medical Research) & Dr Mark Appleyard (Director of Endoscopy, Gastroenterological Services, Royal Brisbane & Women's Hospital)
Associate Researcher: Ms Kellie Stockton (Research Assistant, Burns, Trauma and Critical Care Research Centre)

I have read, and I understand the Participant Information Version 2 dated 26/09/08.

I freely agree to participate in this project according to the conditions in the Participant Information.

I will be given a copy of the Participant Information and Consent Form to keep.

The researcher has agreed not to reveal my identity and personal details if information about this project is published or presented in any public form.

Participant's Name (printed) ……………………………………………………..

Signature ………………………………… Date ………………………………

Name of Witness to Participant's Signature (printed) ……………………………

Signature ………………………………… Date ………………………………

Researcher's Name (printed) ………………………………………………………

Signature ………………………………… Date ………………………………

Note: All parties signing the Consent Form must date their own signature.

Revocation of Consent Form

Full project Title: Can we Alter Risk Factors for Oesophageal Cancer with Exercise?

Principal Researchers: Ms Brooke Winzer (PhD student, Burns, Trauma and Critical Care Research Centre), Dr Jennifer Paratz (Chair, Burns, Trauma and Critical Care Research Centre), Associate Professor David Whiteman (School of Public Health, Dept of Epidemiology, Queensland Institute of Medical Research) & Dr Mark Appleyard (Director of Endoscopy, Gastroenterological Services, Royal Brisbane & Women's Hospital)

Associate Researcher: Ms Kellie Stockton (Research Assistant, Burns, Trauma and Critical Care Research Centre)

I hereby wish to WITHDRAW my consent to participate in the research proposal described above and understand that such withdrawal WILL NOT jeopardise any treatment or my relationship with Royal Brisbane & Women's Hospital.

Participant's Name (printed) …………………………………………………………..

Signature …………………………………… Date …………………………………
