# Supplementary material for: The Feasibility of an Exercise Intervention in Males at Risk of Oesophageal Adenocarcinoma: A Randomized Controlled Trial
Source: PLoS One. 2015 Feb 23;10(2):e0117922. doi: 10.1371/journal.pone.0117922 (PMC4338269; doi:10.1371/journal.pone.0117922)
Supplement: S3 File — (PDF) [file pone.0117922.s004.pdf]

Week:

EPOC Clinical Data Record

EPOC Participant number: ____________ **Date Collected**: ___________ **Time:** _______

**1. Weight:**	**Input value** [ ][ ][ ].[ ] kg		
**2. Height:**	2a [ ][ ][ ].[ ] cm	2b [ ][ ][ ].[ ] cm	**Input value** [ ][ ][ ].[ ]
**3. Waist Circumference:**	3a [ ][ ][ ].[ ] cm	3b [ ][ ][ ].[ ] cm	**Input value** [ ][ ][ ].[ ]
**4. Hip Circumference:**	4a [ ][ ][ ].[ ] cm	4b [ ][ ][ ].[ ] cm	**Input value** [ ][ ][ ].[ ]
**5. Waist to hip ratio:**	**Input value** [ ].[ ][ ]		

Scale: new

5. Contraindications to BIA: N / Y Pacemaker / Electronic Implant :

6a. Hours since last ate:	6b. Hours since last had caffeine:
6c. Hours since last drank alcohol:	6d. Strenuous exercise in last 12hrs:

7. Gender: M **8. Age:** ________

9. BIA Results

1st

**TBW**	L	%
**ECF**	L	%
**ICF**	L	%
**FFM**	kg	%
**FM**	kg	%
**BMI**		

3rd

**TBW**	L	%
**ECF**	L	%
**ICF**	L	%
**FFM**	kg	%
**FM**	kg	%
**BMI**		

2nd

**TBW**	L	%
**ECF**	L	%
**ICF**	L	%
**FFM**	kg	%
**FM**	kg	%
**BMI**		

Average (input values)

**TBW**	L	%
**ECF**	L	%
**ICF**	L	%
**FFM**	kg	%
**FM**	kg	%
**BMI**		

Week:

EPOC Clinical Data Record

EPOC Participant number: ____________ **Date Collected**: ___________ **Time:** _______

10. Modified Shuttle Walk Test *(perform before strength tests)*

a. VO_2 Peak (ml.min.kg)	ml.min.kg
b. VO_2 Peak (L.min)	L.min
c. HR Resting (bpm)	bpm
d. HR Max (bpm)	bpm
e. RPE resting (6-20)	(6-20)
f. RPE Max (6-20)	(6-20)
d. Shuttle level	

Weight entered in metamax:

Distance in metres:

11. One Repetition Maximum

	Bench Press	Leg Press
Max weight lifted (kg)	**(Not including bar)**	**Seat position (no. of holes exposed):**

12. Questionnaires: **Collected** **SPSS**

- Demographic Questionnaire (wk 0 only) ☐ ☐
- Food Frequency Questionnaire ☐ ☐
- Gastro-oesophageal Reflux Disease Impact Scale ☐ ☐
- IPAQ Long PA Questionnaire ☐ ☐

Entered into SPSS: Weight/height/waist ☐ **BIA** ☐ **MSWT** ☐ **1RM** ☐

Name:
Date:
