# Supplementary material for: The Feasibility of an Exercise Intervention in Males at Risk of Oesophageal Adenocarcinoma: A Randomized Controlled Trial
Source: PLoS One. 2015 Feb 23;10(2):e0117922. doi: 10.1371/journal.pone.0117922 (PMC4338269; doi:10.1371/journal.pone.0117922)
Supplement: S1 Protocol — (PDF) [file pone.0117922.s005.pdf]

**Can We Alter Risk Factors For Oesophageal Cancer With Exercise?**

DESIGN

A prospective, randomised controlled trial will be implemented.

SUBJECTS

*Inclusion Criteria*

Participants will be overweight males (BMI 25-34.99 kg/m2)

Participants must also be Brisbane residents, aged between 18 and 70 years old with BE diagnosed via endoscopy. Furthermore, participants must be sedentary, performing less than 60 minutes of moderate intensity, structured exercise per week.

*Exclusion Criteria*

Potential participants with cardiac disease; uncontrolled hypertension or hypotension; diabetes; orthopaedic injuries; neurological, respiratory, haematological or inflammatory conditions; or any other serious impairment that would impede their ability to perform the exercise intervention will be excluded. Participants with a past history of cancer within 5 years will also be excluded. Participants with a BMI >35 $kg/m^2$ will be excluded due to safety concerns. Participants who have already commenced an exercise program or diet in the past 6 months will also be excluded as will participants who have lost or gained more than 5kg in the past 6 months.

RECRUITMENT

Participants will be recruited via the following avenues:

- The gastroenterology clinic at the Royal Brisbane & Women's Hospital.
- A database of people with BE whom have previously participated in epidemiological studies at the Queensland Institute of Medical Research. A member of the research team who previously worked with these participants (A/Prof Whiteman) will contact them for permission to be approached by our research team.
- Via the rooms of private gastroenterologists and the endoscopy suite at The Wesley Hospital.
- Gastroenterology department at the Princess Alexandra Hospital
- Gastroenterology department at Greenslopes Private Hospital
- Private clinics of Gastro-Intestinal Endoscopy Services (GIE)
- Gastroenterology department at The Prince Charles Hospital

GROUP ALLOCATION

Participants will be allocated to one of two groups via computerised randomisation. The randomisation sequence will be protected by an electronic password. Group allocation will be concealed. Baseline measures will then be taken on all participants.

EXERCISE INTERVENTION

Participants will complete 1hr exercise sessions, at moderate intensity (60-70% of maximum heart rate), five days/week for 24 weeks. Each session will combine cardiovascular training with resistance training. The program is consistent with the American College of Sports Medicine guidelines to attain weight loss[1].

Participants will perform one supervised exercise session per week at the Royal Brisbane & Women's Hospital (RBWH) or The Wesley Hospital under the direction of a physiotherapist (Brooke Winzer). Participants will perform the remaining four exercise sessions independently at a local gym or workplace gym as they are essentially healthy and do not require constant monitoring. Participants will document the details of their independent sessions in an exercise diary which will be reviewed by Brooke Winzer on a weekly basis at the supervised session. Progression of exercises will also occur during the supervised session.

Cardiac Rehab Manager Sandi McKellar has agreed to provide space in the physiotherapy gym at The Wesley hospital for the intervention to take place.

Exercise intensity will be measured via the unmodified (6-20) BORG scale[2]. Participants will be familiarised with the scale on their initial supervised exercise session and will be instructed to maintain their rate of perceived exertion (RPE) between "fairly light" and "somewhat hard" or "steady pace". During the initial session, a pulse oximeter will be used to ensure participant's heart rates are maintained between 60-70% of maximum and correlate with the desired RPE. A variety of aerobic exercises will be prescribed to maintain participant motivation and interest such as treadmills, exercise bikes and seated rowing.

Resistance training will include: one-two sets of 8-15 repetitions performed on the major muscle groups. Free weights and machine weights will be used. Exercises will include: bench press, seated row, leg press, lunges, shoulder press, assisted chin ups and assisted dips. Resistance will be set at achieving muscular fatigue between 12-15 repetitions initially and then 8-10 repetitions after 8 weeks of training. Resistance will therefore progressively increase as the subjects enhance their strength.

CONTROL GROUP

To make the groups as similar as possible the control group will attend the RBWH or The Wesley Hospital once a week to perform stretches under the instruction of Brooke Winzer. The control group will also be instructed to perform the stretching program at home, four times per week. Stretching is not strenuous enough to burn significant energy to attain fat loss and will act as a sham intervention.

Participants allocated to the control group will receive a rubber mat so they can perform the stretching program comfortably at home.

The control group will be instructed not to commence a new exercise regime during the study other than the prescribed stretching program. Therefore participants will remain sedentary.

At the conclusion of the study the control group will be offered a 3 month gym membership in recognition of their involvement in the study. Offering part of the intervention to control participants at the conclusion of the study is becoming standard practice among cancer prevention clinical trials to increase retention and ultimately improve methodological quality.

PRIMARY OUTCOME MEASURES

A research nurse will perform the collection of all blood samples. Fasting blood samples of 30ml will be obtained by venipuncture from the antecubital vein. Blood samples will be taken at least 24hrs since the most recent exercise session. Samples will be centrifuged, serum aliquoted for subsequent analyses and stored at -70ºC. The analysis of all blood samples will be performed by blinded researchers. All blood analysis will be performed on serum.

1. Leptin and Adiponectin serum concentrations will be measured by a radioimmunoassay (RIA) from Linco Research Inc. (Australian Laboratory Services, Sydney, Australia). The intra-assay and inter-assay co-efficients of variability are less than 5%. All samples will be analysed in duplicate.

2. CRP, and IL-6, TNF-alpha serum concentrations will also be analysed using RIAs (Linco Research Inc.,Australian Laboratory Services, Sydney, Australia). All samples will be analysed in duplicate. The intra-assay and inter-assay co-efficients of variability are less than 5%.

3. Glucose and insulin serum concentrations will be measured in duplicate via a fasting blood test and analysed using RIAs (Linco Research Inc., Australian Laboratory Services, Sydney, Australia). The coefficients of variation for all assays are <10%, and for insulin, <5%. Insulin resistance will be determined using the reciprocal index of homeostasis model assessment (HOMA-IR) and QUICKI index. Both measures calculate ratios of fasting insulin:glucose concentrations using different algorithms, and have been shown to be reproducible and valid measures of insulin resistance[3,4].

All primary outcome measures will be taken at the following time points:

- Once participants have enrolled, before the intervention begins (0 weeks).
- Half way through the intervention (12 weeks).
- At the conclusion of the intervention (24 weeks).

SECONDARY OUTCOME MEASURES

1. Body fat will be measured using bioelectrical impedance analysis (BIA), body mass index (BMI), and the ratio of waist to hip circumference (waist:hip ratio).

    - *Bioelectrical Impedance Analysis:* BIA will be measured using a SEAC Multi-Frequency Bioelectrical Impedance Analyser (SEAC, Brisbane). BIA measures will be taken in triplicate and the results averaged[5]. Typical coefficients of variation for within a measurement session range from 0.3 to 3% [5].

    - *BMI* will be also calculated (weight/height$^2$) as it is a reasonable measure of global adiposity.

    - *Weight*: measured to nearest 0.1 kg in light clothing without shoes on a calibrated scale (Lindel balance scale, Samhald, Klippan Sweden) with an empty bladder.

    - *Waist Circumference*: The measuring tape is placed in a horizontal plane around the abdomen immediately above the top of the right iliac crest. The plane of the tape is parallel to the floor and the tape is snug, but does not compress the skin. The measurement is made at a normal minimal respiration (NHANES III protocol)[6].

    - *Hip Circumference*: measured as the maximum diameter at the greater trochanter with a horizontal tape. The data will be used to calculate BMI and waist:hip ratio.

2. Cardiovascular Fitness. The Modified Shuttle Walk Test (MSWT) will be used to predict $VO_2$peak. The test involves walking between two markers spaced 10 metres apart at a pace set by an audio tape. The MSWT progressively gets more difficult as the speed of the tape gradually increases, eventually requiring the person to run between the markers.

   A small training or learning effect has been shown between the first and second trial, therefore participants will perform the test twice with a 30minute rest in between[7].

   The MSWT has been shown to strongly correlate with $VO_2$max during treadmill testing (r=0.88) in patients with airflow obstruction[8], however we will confirm this in our population by monitoring participants with a Cortex Metamax 3 portable metabolic analyser (Cortex:biophysik, GMbH, Germany) during the test. $VO_2$peak will be confirmed using established physiological criteria and includes a heart rate near maximal for age, the anaerobic threshold (V-slope technique), oxygen uptake reaching a plateau with increasing work rate and a RPE of 20/20[9]. The test is ceased when either: $VO_2$peak is obtained, the person cannot keep up with the audio tape or the person cannot continue due to breathlessness or fatigue, and requests to stop. Participants will only be monitored with the metabolic analyser during the second trial. The first trial will be used as a practice.

   Given that our population is sedentary and overweight or obese, we feel maximal exertion will be reached during this test as it combines a wide range of exercise intensities from slow walking (0.50m/s) to fast running (2.37m/s). The test will be performed a second time after a 30 minute rest. A recent study of overweight, untrained middle aged males required 34 participants in each group to detect a significant improvement in $VO_2$peak in the exercise group[10].

3. Muscular Strength. To measure muscular strength, a one-repetition maximum on each resistance exercise will be taken. One-repetition maximum is the maximum weight a person can lift only one time with correct technique. It has shown to be a reliable and valid measure of strength in middle aged, untrained adults[11].

4. Gastro-oesophageal reflux symptom frequency and duration will be measured using a validated questionnaire[12]. As gastro-oesophageal reflux severity is associated with obesity[13] and increased oesophageal AC risk[14] we are interested in the effect the exercise intervention may have on this disorder.

All secondary outcomes will be measured on enrolment (0 weeks), midpoint (12 weeks) and at the conclusion of the intervention (24 weeks).

OTHER MEASURES

- Physical Activity Diary: Participants will document all exercise performed on a daily basis for the duration of the study.

- Food Frequency Questionnaire. To ensure a stable and comparable diet between the two groups, participants will complete a food frequency questionnaire at the commencement of the study (week 0), midpoint (week 12) and at the conclusion of the study (week 24).

- International Physical Activity Questionnaire (2002). This short self administered questionnaire will provide additional information regarding sedentary time, incidental and leisure time physical activity. Such information will be important when interpreting the results of this study.

REFERENCES

1. Pollock ML, et al: ACSM position stand: The recommended quantity and quality of exercise for developing and maintaining cardiorespiratory and muscular fitness and flexibility in healthy adults. Medicine & Science in Sports and Exercise 1998;30(6);975-991.
2. Mielke M, et al: The development of rating of perceived exertion-based tests of physical working capacity. J Strength Cond Res 2008; 22:293-302.
3. Mathews DR, et al: Homeostasis model assessment: insulin resistance and beta-cell function from fasting plasma glucose and insulin concentrations in man. Diabetologia 1985;28:412-9.
4. Singh SJ et al: Development of a shuttle walking test of disability in patients with chronic airways obstruction. *Thorax* 1992; 47:1019-1024.
5. Singh SJ et al: Comparison of oxygen uptake during a conventional treadmill test and the shuttle walking test in chronic airflow limitation. *Eur Respir J* 1994; 7: 2016-2020.
6. Katz A, et al: Quantitative insulin sensitivity check index: a simple, accurate method for assessing insulin sensitivity in humans. J Clin Endocrinol Metab 2000; 85:2402-10.
7. Cornish BH, et al: The Evaluation of multiple frequency bioelectrical impedance analysis for the assessment of body water volumes in healthy humans Eur J Clin Nutr 1996; 50; 159-164
8. U.S Department of Health and Human Services: NHANES III Anthropometric Procedures Video. Washington, D.C, Public Health Service, 1996
9. Fletcher GF, et al: Exercise standards for testing and training: a statement for healthcare professionals from the American heart Association. Circulation 2001; 104: 1694-1740
10. Andersenn SA,et al:Combined diet and exercise intervention reverses the metabolic syndrome in middle-aged males. Scand J Med Sci Sports 2007;17:687-695.
11. Levinger I, et al: The reliability of the 1RM strength test for untrained, middle-aged individuals. J Sci Med in Sport (In Press)
12. Jones R, Coyne K, & Wiklund I: The Gastro-oesophageal Reflux Disease Impact Scale: A patient management tool for primary care. *Aliment Pharmacol Ther* 2007; 25(12): 1451-1459.
13. El-Serag HB, et al: Obesity increases oesophageal acid exposure. *Gut* 2007; 56:749-755.
14. Whiteman DC, et al: Combined effects of obesity, acid reflux and smoking on the risk of adenocarcinomas of the oesophagus. *Gut* 2008;57:173-180.
