# Supplementary material for: The Feasibility of an Exercise Intervention in Males at Risk of Oesophageal Adenocarcinoma: A Randomized Controlled Trial
Source: PLoS One. 2015 Feb 23;10(2):e0117922. doi: 10.1371/journal.pone.0117922 (PMC4338269; doi:10.1371/journal.pone.0117922)
Supplement: S1 Table — (DOCX) [file pone.0117922.s006.docx]

**Table S1: Physical activity^a^ and dietary outcomes at baseline and week-24 comparing participants in the exercise group (n=16) and control group (n=15).**

|  | Baseline | 24-Weeks | Change from baseline to 24-weeks | | Intervention effect  (Exercise – Control) | |
| --- | --- | --- | --- | --- | --- | --- |
|  | Mean (SD) | Mean (SD) | Mean (95%CI) | % change | Mean (95%CI) | p-value^b^ |
| Recreational activity (MET-h/wk) |  |  |  |  |  |  |
| Exercise group | 6.0 (1.2, 13.3) ^c^ | 41.7 (24.8, 51.3) ^c^ | 29.5 (21.6, 37.4) | 331.5 | 27.3 (15.9, 38.7) | < 0.01 |
| Control group | 4.0 (0.0, 9.9) ^c^ | 8.0 (3.3, 16.9) ^c^ | 2.2 (-5.9, 10.4) | 36.1 |  |  |
| Occupational activity (MET-h/wk) |  |  |  |  |  |  |
| Exercise group | 0.0 (0.0, 12.6)^c^ | 6.7 (0.0, 31.5)^c^ | 9.6 (-15.7, 34.9) | 75.6 | -12.2 (-48.8, 24.3) | 0.49 |
| Control group | 1.7 (0.0, 15.7)^c^ | 14.9 (0.0, 49.1)^c^ | 21.8 (-4.3, 47.9) | 89.7 |  |  |
| Household activity (MET-h/wk) |  |  |  |  |  |  |
| Exercise group | 13.0 (1.9, 32.2)^c^ | 20.8 (8.0, 66.0)^c^ | 10.8 (-4.0, 25.6) | 43.5 | 5.0 (-16.5, 26.7) | 0.63 |
| Control group | 8.7 (3.6, 34.0)^c^ | 19.5 (11.5, 52.5)^c^ | 5.7 (-10.1, 21.6) | 26.0 |  |  |
| Sitting time (h/wk) |  |  |  |  |  |  |
| Exercise group | 52.0 (18.2) | 37.4 (12.8) | -11.8 (-17.2, -6.4) | -22.7 | 0.0 (-7.8, 7.9) | 0.99 |
| Control group | 43.3 (17.8) | 34.1 (12.5) | -11.8 (-17.3, -6.2) | -27.3 |  |  |
| Total energy intake (kcal/day) |  |  |  |  |  |  |
| Exercise group | 2259 (593) | 2126 (414) | -130 (-375, 116) | -5.7 | -380.1 (-734.2, -26.0) | 0.04 |
| Control group | 2183 (587) | 2479 (700) | 249 (-4, 503) | 11.4 |  |  |

^a^ Measured using the International Physical Activity Questionnaire.

^b^ Change in exercise group versus change in control group, adjusted for baseline value (ANCOVA).

^c^ Data are median (25^th^, 75^th^ percentile)
