# Supplementary material for: The Feasibility of an Exercise Intervention in Males at Risk of Oesophageal Adenocarcinoma: A Randomized Controlled Trial
Source: PLoS One. 2015 Feb 23;10(2):e0117922. doi: 10.1371/journal.pone.0117922 (PMC4338269; doi:10.1371/journal.pone.0117922)
Supplement: S2 Table — (DOCX) [file pone.0117922.s007.docx]

**Table S2. Obesity-related hormone outcomes at baseline and week-12 comparing participants in the exercise group (n=15) and control group (n=16).**

|  | Baseline | 12-Weeks | Change from baseline to  12-weeks | | Intervention effect  (Exercise – Control) | |
| --- | --- | --- | --- | --- | --- | --- |
|  | Mean (SD) | Mean (SD) | Mean (95%CI) | % change | Mean (95% CI) | p-value^a^ |
| Leptin (ng/mL) |  |  |  |  |  |  |
| Exercise group | 11.7 (7.7) | 10.1 (7.4) | -2.1 (-4.4, 0.3) | -17.9 | -2.82 (-6.1,0.4) | 0.09 |
| Control group | 13.0 (9.2) | 13.7 (10.0) | 0.7 (-1.5, 2.9) | 5.4 |  |  |
| Total Adiponectin (μg/mL) |  |  |  |  |  |  |
| Exercise group | 6.5 (2.7) | 4.8 (1.6) | -1.6 (-2.1,-1.0) | -24.6 | -0.15 (-0.9,0.6) | 0.70 |
| Control group | 5.9 (2.8) | 4.6 (1.8) | -1.4 (-2.0,-0.9) | -23.7 |  |  |
| HMW adiponectin (μg/mL) |  |  |  |  |  |  |
| Exercise group | 2.8 (1.5) | 1.8 (0.9) | -0.8 (-1.1, -0.4) | -28.6 | -0.30 (-0.8,0.2) | 0.22 |
| Control group | 2.1 (1.4) | 1.7 (1.1) | -0.5 (-0.8, -0.1) | -23.8 |  |  |
| IL-6 (pg/mL) |  |  |  |  |  |  |
| Exercise group | 4.3 (3.4) | 4.9 (3.4) | - | - | 1.12 (0.73, 1.71)^b^ | 0.59 |
| Control group | 4.3 (5.2) | 5.1 (6.3) | - | - |  |  |
| TNF-α (pg/mL) |  |  |  |  |  |  |
| Exercise group | 7.0 (2.8) | 8.0 (3.2) | 1.0 (0.2, 1.8) | 13.7 | 0.91 (-0.3,2.1) | 0.13 |
| Control group | 6.5 (1.9) | 6.5 (2.5) | 0.1 (-0.8, 0.9) | 0.8 |  |  |
| CRP (mg/L) |  |  |  |  |  |  |
| Exercise group | 3.5 (6.3) | 2.8 (2.4) | -0.8 (-2.0, 0.5) | -22.8 | 0.80 (-0.9,2.5) | 0.34 |
| Control group | 3.5 (5.1) | 2.0 (2.1) | -1.6 (-2.8,-0.4) | -45.7 |  |  |
| HOMA |  |  |  |  |  |  |
| Exercise group | 2.6 (1.1) | 1.9 (0.7) | -0.9 (-1.5, -0.4) | -35.4 | -0.47 (-1.3,0.3) | 0.24 |
| Control group | 3.6 (2.3) | 3.0 (2.1) | -0.5 (-1.0, 0.1) | -12.5 |  |  |

HMW, high molecular weight; IL-6, interleukin-6; TNF-α, tumour necrosis factor-alpha; CRP, C-reactive protein; HOMA, homeostasis model assessment.

^a^ Change in exercise group versus change in control group, adjusted for baseline value (ANCOVA).

^b^ Backtransformed natural log; expressed as relative ratio
