# Supplementary material for: The Feasibility of an Exercise Intervention in Males at Risk of Oesophageal Adenocarcinoma: A Randomized Controlled Trial
Source: PLoS One. 2015 Feb 23;10(2):e0117922. doi: 10.1371/journal.pone.0117922 (PMC4338269; doi:10.1371/journal.pone.0117922)
Supplement: S3 Table — (DOCX) [file pone.0117922.s008.docx]

**Table S3. Median (25^th^, 75^th^ percentile) of obesity-related hormones at baseline, week 12 and week 24.**

|  | Baseline | 12-Weeks | 24-Weeks |
| --- | --- | --- | --- |
| Leptin (ng/mL) |  |  |  |
| Exercise group | 10.4 (4.5,17.5) | 6.7 (5.9,13.3) | 7.5 (4.6, 12.9) |
| Control group | 10.0 (6.0,20.0) | 9.2 (6.6,20.5) | 9.3 (6.7,17.8) |
| Total Adiponectin (μg/mL) |  |  |  |
| Exercise group | 5.5 (4.4,7.7) | 4.4 (3.4,6.0) | 5.2 (3.3,6.1) |
| Control group | 5.0 (4.1,7.6) | 4.3 (3.4,5.9) | 4.2 (3.6,4.8) |
| HMW adiponectin (μg/mL) |  |  |  |
| Exercise group | 2.6 (1.3,3.7) | 1.75 (1.0,2.5) | 2.3 (1.0,3.0) |
| Control group | 1.5 (1.2, 3.3) | 1.3 (0.9, 2.2) | 1.4 (1.2,2.0) |
| IL-6 (pg/mL) |  |  |  |
| Exercise group | 3.9 (1.8,6.0) | 3.1 (2.3,7.2) | 1.8 (1.4,6.9) |
| Control group | 2.3 (1.1,5.5) | 1.6 (1.0,6.9) | 2.2 (0.9,10.3) |
| TNF-α (pg/mL) |  |  |  |
| Exercise group | 6.8 (5.4,8.0) | 7.0 (5.1, 11.1) | 5.9 (5.5, 8.9) |
| Control group | 6.4 (5.1,7.3) | 6.1 (5.0,7.2) | 5.9 (5.2,7.0) |
| CRP (mg/L) |  |  |  |
| Exercise group | 1.0 (0.6, 3.8) | 1.9 (1.3, 3.6) | 1.5 (0.6,3.0) |
| Control group | 1.8 (0.9,3.6) | 1.0 (0.5,3.1) | 0.9 (0.5, 1.3) |
| HOMA |  |  |  |
| Exercise group | 2.5 (1.9,2.8) | 1.9 (1.2,2.4) | 2.1 (1.5, 2.4) |
| Control group | 3.3 (1.7,5.0) | 2.8 (1.5,3.5) | 1.8 (1.2,4.9) |

HMW, high molecular weight; IL-6, interleukin-6; TNF-α, tumour necrosis factor-alpha; CRP, C-reactive protein; HOMA, homeostasis model assessment.
