# Supplementary material for: The Feasibility of an Exercise Intervention in Males at Risk of Oesophageal Adenocarcinoma: A Randomized Controlled Trial
Source: PLoS One. 2015 Feb 23;10(2):e0117922. doi: 10.1371/journal.pone.0117922 (PMC4338269; doi:10.1371/journal.pone.0117922)
Supplement: S4 Table — (DOCX) [file pone.0117922.s009.docx]

**Table S4. Body composition, fitness, strength and gastro-oesophageal reflux outcomes at baseline and week-12 comparing participants in the exercise group and control group.**

|  |  | Baseline | 12-Weeks | Change from baseline to  12-weeks | | Intervention effect  (Exercise – Control) | |
| --- | --- | --- | --- | --- | --- | --- | --- |
|  | n | Mean (SD) | Mean (SD) | Mean (95%CI) | % change | Mean (95%CI) | p-value^a^ |
| Waist circumference (cm) |  |  |  |  |  |  |  |
| Exercise group | 16 | 105.3 (8.2) | 102.7 (8.8) | -2.7 (-4.9, -0.6) | -2.6 | -2.46 (-5.5,0.6) | 0.11 |
| Control group | 16 | 104.8 (10.6) | 104.6 (9.7) | -0.3(-2.4, 1.9) | -0.3 |  |  |
| Weight (kg) |  |  |  |  |  |  |  |
| Exercise group | 16 | 94.7 (10.5) | 93.3 (11.2) | -1.0 (-2.3, 0.3) | -1.1 | -0.55 (-2.4,1.3) | 0.56 |
| Control group | 16 | 92.4 (9.6) | 91.9 (9.3) | -0.5 (-1.8, 0.9) | -0.5 |  |  |
| Fat mass (kg) |  |  |  |  |  |  |  |
| Exercise group | 16 | 22.0 (6.1) | 19.5 (5.6) | -2.8 (-4.5, -1.1) | -12.7 | -2.10 (-4.5,0.3) | 0.08 |
| Control group | 16 | 21.6 (8.7) | 21.0 (5.6) | -0.7 (-2.4, 1.0) | -3.2 |  |  |
| Lean Mass (kg) |  |  |  |  |  |  |  |
| Exercise group | 16 | 72.6 (10.3) | 73.6 (10.5) | 2.0 (-0.2, 4.2) | 2.8 | 2.09 (-1.0,5.2) | 0.17 |
| Control group | 16 | 70.9 (7.6) | 70.9 (6.5) | -0.1 (-2.3, 2.1) | -0.1 |  |  |
| VO_2_ peak (mL/min/kg) |  |  |  |  |  |  |  |
| Exercise group | 15 | 26.1 (8.2) | 30.5 (7.4) | 5.1 (1.9, 8.3) | 19.5 | 2.31 (-2.3,6.9) | 0.31 |
| Control group | 15 | 26.8 (7.5) | 29.6 (7.8) | 2.8 (-0.4, 6.0) | 10.4 |  |  |
| Bench press (kg) |  |  |  |  |  |  |  |
| Exercise group | 16 | 37.1 (14.3) | 45.3 (11.8) | 9.3 (6.0, 12.6) | 25.1 | 7.26 (2.5,12.0) | <0.01 |
| Control group | 15 | 35.0 (13.3) | 36.7 (12.1) | 2.1 (-1.4, 5.5) | 6.0 |  |  |
| Leg Press (kg) |  |  |  |  |  |  |  |
| Exercise group | 16 | 136.5 (35.2) | 163.6 (35.2) | 28.9 (18.8, 38.9) | 21.2 | 17.87 (3.6,32.1) | 0.02 |
| Control group | 16 | 140.2 (46.6) | 150.8 (45.5) | 11.0 (1.0, 21.0) | 7.8 |  |  |
| Gastro-oesophageal reflux^b^ |  |  |  |  |  |  |  |
| Exercise group | 16 | 10.8 (1.0) | 11.1 (0.9) | 0.3 (-0.1, 0.7) | 2.8 | 0.38 (-0.6,0.6) | 0.89 |
| Control group | 15 | 10.8 (1.5) | 11.1 (1.0) | 0.3 (-0.2, 0.7) | 2.8 |  |  |

^a^ Change in exercise group versus change in control group, adjusted for baseline value (ANCOVA).

^b^ Measured using Gastro-oesophageal reflux disease impact scale.
